# The social disorganization of eating: a neglected determinant of the Australian epidemic of overweight/obesity

**DOI:** 10.1186/s12889-019-6768-3

**Published:** 2019-06-03

**Authors:** Michael Bittman, Eimear Cleary, Charlotte Wilkinson-Bibicos, Jonathan Gershuny

**Affiliations:** 1 Sociology, BCSS, University of New England, Armidale, Australia; 2 Department of Global Health, Australian National University, Canberra, Australia; 3 Department of English, University of Sydney, Sydney, Australia; 4 Centre for Time Use Research, Sociology, University of Oxford, Oxford, UK

**Keywords:** Social organization of eating, Eating occasions, Mealtimes, Mindless eating

## Abstract

**Background:**

Over the last 150 years, advanced economies have seen the burden of disease shift to non-communicable diseases. The risk factors for these diseases are often co-morbidities associated with unhealthy weight. The prevalence of overweight/obesity among adults in the advanced countries of the English-speaking world is currently more than two-thirds of the adult population. However, while much attention has concentrated on changes in diet that might have provoked this rapid increase in unhealthy weight, changes in patterns of eating have received little attention.

**Methods:**

This article examines a sequence of large-scale, time use surveys in urban Australia stretching from 1974 to 2006. The earliest survey in 1974 (conducted by the Cities Commission) was limited to respondents aged between 18 and 69 years, while the later surveys (by the Australian Bureau of Statistics) included all adult (15 years of age or over) living private dwellings. Since time use surveys capture every activity in a day, they contain much information about mealtimes and the patterns of eating. This includes duration of eating, number of eating occasions and the timing of eating. Inferential statistics were used to test the statistical significance of these changes and the size of the effects.

**Results:**

The eating patterns of urban Australian adults have changed significantly over a 32-year period and the magnitude of this change is non-trivial. Total average eating time as main activity has diminished by about a third, as have eating occasions, affecting particularly luncheon and evening meals. However, there is evidence that eating as secondary activity that accompanies another activity is now almost as frequent as eating at mealtimes. Moreover, participants seem not to report it.

**Conclusions:**

Contemporary urban Australians are spending less time in organized shared meals. These changes have occurred the over same period during which there has been a public health concern about the prevalence of unhealthy weight. Preliminary indications are that societies that emphasize eating *as a commensal, shared activity through maintaining definite, generous lunch breaks and prioritizing eating at mealtimes, achieve* better public health outcomes. This has implications for a strategy of health promotion, but to be sure of this we need to study countries with these more socially organized eating patterns.

**Electronic supplementary material:**

The online version of this article (10.1186/s12889-019-6768-3) contains supplementary material, which is available to authorized users.

## Background

Public health originated in the study of epidemics of communicable disease in the nineteenth century. In the twenty-first century the major burden of disease has shifted to non-communicable diseases. Murray and Lopez calculated that, among developed economies, 83% of the burden of disease in the year 2020 will be attributable to non-communicable diseases [1]. The major task of public health then becomes one of persuading people to change behaviours. These twenty-first century ‘killer’ diseases are sometimes known as ‘lifestyle’ diseases because there are strong associations between certain daily practices and so-called ‘risk factors’. Diet quickly became a focus of research [2], but there has been little research devoted to studying how social patterns of eating are related to the overweight/obesity epidemic. This paper uses historical data available through repeated time-use surveys in a developed English-speaking country – Australia – to show how changes in the social organization of eating are associated with increased rates of adult overweight/obesity.

Between the late 1980s and 2010 there was a steep increase in overweight/obese body mass index (BMI) patterns in English-speaking countries, including Australia, from a starting point where more than half the adult population was already heavier than healthy weight [3, 4]. These societies would reach rates of two-thirds or even three-quarters of the adult population by the end of the second decade of the twenty-first century. In contrast, Mediterranean countries, Switzerland and the Republic of Korea have not yet reached the levels of BMI in the English-speaking countries at the beginning of the period (although the proportions of overweight/obese people have also been increasing in these latter countries).

Starting with the work led by Carlos Monteiro in Brazil [5–12], recent literature on epidemiological nutrition has suggested that eating over three decades has shifted to ready-to-consume food, also described as ‘ultra-processed foods’. Monteiro and his colleagues classify food into three groups: Group 1 consists of ‘minimally processed foods … whole foods that have been submitted to some process that does not substantially alter the nutritional properties of the original foods’; Group 2 involves ‘substances extracted from whole foods’, such as oils, fats, flours, pastas, starches and sugars, that are traditionally used ‘in the domestic preparation and cooking of dishes mainly made up of fresh and minimally processed foods’; Group 3 involve ‘ultra-processed foods [that] are basically confections of group 2 ingredients, typically combined with sophisticated use of additives, to make them edible, palatable, and habit-forming’ [5]. There has been a trend away from less processed foods and towards ultra-processed foods, a trend most evident in the advanced societies of the English-speaking world. For example, ready-to-consume products in the UK account for 63.4% of all calories consumed, compared to 27.7% in Brazil, although this has been rapidly increasing [11, 13]. Current conventional food classifications are based on types of nutrients or foods, and discussions of food, nutrition and health generally also ignore ‘the ways in which food processing affects patterns of purchase, use and consumption’ [8].

Claude Fischler, the French anthropologist/sociologist, makes some similar points to Monteiro and colleagues. In addition, he has argued that ‘in most if not all societies on the planet, eating is done in a social context’. Furthermore, ‘the procurement, distribution and sharing of food and the social regulation thereof are the basis for much of social organization in human societies’. However, he comments, ‘most campaigns and public policies so far have been based on implicit assumptions, in particular that eating is just another form of individual, private consumption’. The hope is that health education will shape individual food preferences. Fischler argues that this has ‘more drawbacks than benefits’ because it individualizes and privatizes eating. It leads to a ‘nutritional cacophony’: questionable diets, anxiety, eating disorders and, most importantly, ‘no reduction in the prevalence of obesity’, especially among those categories of the population where improvement is most desired. This private individual approach neglects the ‘long unsuspected benefits associated with the sharing of food in the common meal [14]. In other words, the social organization of eating may be as important as, or even more important than, the macro-nutrients or the amount of processing in the food consumed. This paper uses historical data available through repeated time-use surveys in a developed English-speaking country – Australia – to show how changes in the social organization of eating are associated with increased rates of adult overweight/obesity.

## Methods

The studies used in this analysis involved respondents completing a time-diary of activities undertaken during a designated day. The diary has rows for the hours of the day, divided into 10-min intervals. Respondents report their ‘main activity’ and whether they were doing something else at the same time, together with the context of the activity – the location of the activity, and the company present.

### Availability of data and materials

The main source of data is the Multinational Time-Use-Study (MTUS) [15] encompassing over 60 datasets from 25 countries of patterns of time-use from 1992 to 2006. For longer-run changes in Australian eating patterns, this data is supplemented by information from the 1987 Pilot Study of Time-Use Sydney [16], and the metropolitan component of the Australian Cities Commission Time-Use Survey 1974 [17]. Australia has always been a highly urbanized country. In 1973, 65% of the Australian population lived in a capital city, and by 2013, this proportion had marginally increased to 66%. Therefore, despite this limitation, the resulting data covers most of the population and closely approximates national trends. However, the coding of ‘breaks at work’ in the 1987 survey did not distinguish between ‘eating’ and other activities undertaken at this time, so the datasets have been adjusted to combine time spent ‘eating’ with time spent in ‘breaks at work’. Additional file 1: Table S1 gives details for each study, including sample size for those resident in major urban centres.

Time-use studies are descended from the standardized activity codes and methods of administration developed for the 1965 UNESCO-sponsored study of 12 nations [18], although activities are not identical across all surveys. New activities arise (e.g. computing) and others become uncommon (e.g. fetching water or making jam). But the surveys are easily harmonized to facilitate direct comparisons of change over time.

### Analysis

The main dependent variables are time spent ‘eating’ (and ‘breaks at work’) and time spent in ‘preparation of food and drink’ at home.

An ordinary least squares procedure was used to calculate a linear trend line (sometimes called a ‘line of best fit’) of the changes is time spent eating. Inferential statistics (t-test for independent samples were used to determine if this change was unlikely to have occurred by chance. Cohen’s *d* was used to judge the magnitude of this change, since large samples can produce trival effect sizes which are statistically significant.

Because the time spent in (main) activities cannot be greater than the number of minutes in a day (i.e. 1440), analysing time-use data typically uncovers trends in the mean time spent in any activity. This inflexible upper limit makes it possible to show time ‘displacement’. An increase in the time spent in some activities means a decrease in the time spent in other activities (and vice versa) [19].

As well as trends in the displacement of time from one activity to another, the analysis studies trends in the average number of daily eating events. This summarizes information about how often adult Australians ate during the day. Further analysis adds a temporal dimension of the times when Australians ate, and the social context (coordinating social meal times with significant others). This synchronization of eating is a key feature of most societies, and provides some insight into the processes of the social coordination of eating. To map this, the analysis uses the ‘tempogram’ from the ‘Szalai study’ of 1965 [18], a diagram depicting the proportion of the population undertaking a particular activity at a particular time of day. Consuming food is a universal activity, but its preparation is culturally specific. Here, Fischler’s concept of commensality is relevant, as it refers to the ways in which groups socially organize their eating habits [14].

Analysis of the time men and women spent in food and drink preparation at home is relevant to changes in the social organization of eating. It allows us to study the association between changes in the social position of women and the production of meals.

## Results

### Historical change in the average duration of Australian eating

**Fig. 1:**
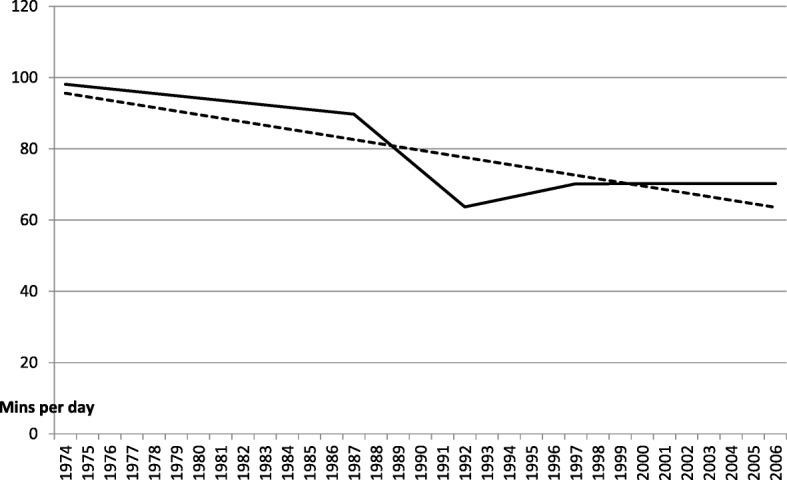
Urban Australian: Two phases in the trend in average daily eating

In Fig. 1, the dashed line is a trend line (sometimes called a ‘line of best fit’). It reduces the fluctuations in eating time to a single linear slope. The line begins in 1974 at 98 min a day and reduces to around 63–70 min per day by 2006, a fall of roughly a third over the five historical points in time, spread over 32 years. This reflects a significant switch to a shorter duration of reported eating time. The hypothesis that daily eating time used to be significantly longer than more recent surveys suggest, is supported by robust independent *t*-tests, as shown in Table 1.

**Table 1:** Details of independent samples *t*-test and Cohen’s *d* scores

Independent samples test											
		Levene’s Test for Equality if Variances		t-test for Equality of Means							
		F	Sig	t	df	Sig (2-tailed)	Mean Difference	Std. Error Difference	Interval of the Difference		Cohen’sd
									Lower	Upper	
Eat at home or work break	Equal variance assumed	2.7	0.00	13.70	94,411	0.00	28.64	2.09	24.54	32.73	
	Equal variances not assumed			16.60	908.23	0.00	28.64	1.72	25.25	32.02	0.58

Results derived from large datasets can be often be statistically significant although the magnitude of effect might be trivial. A widely used test for the magnitude of the effect is Cohen’s *d*. The Cohen’s *d* for the change in Australia in the daily duration of meals and snacks was 0.58 between 1974 and 2006. According to Cohen’s own guidelines (1992), this change in effect size is substantial enough to be rated as ‘medium’. This measure strongly suggests that the historical change captured by this analysis is non-trivial [20].

As shown in the solid line in Fig. 1, this change occurred in two historical phases. In the first, there was a progressive reduction in duration between the early 1970s and 1992. In the second phase, after 1992, the average duration of eating time plateaus.

### Change in the number of daily eating events (in Australia)

**Fig. 2:**
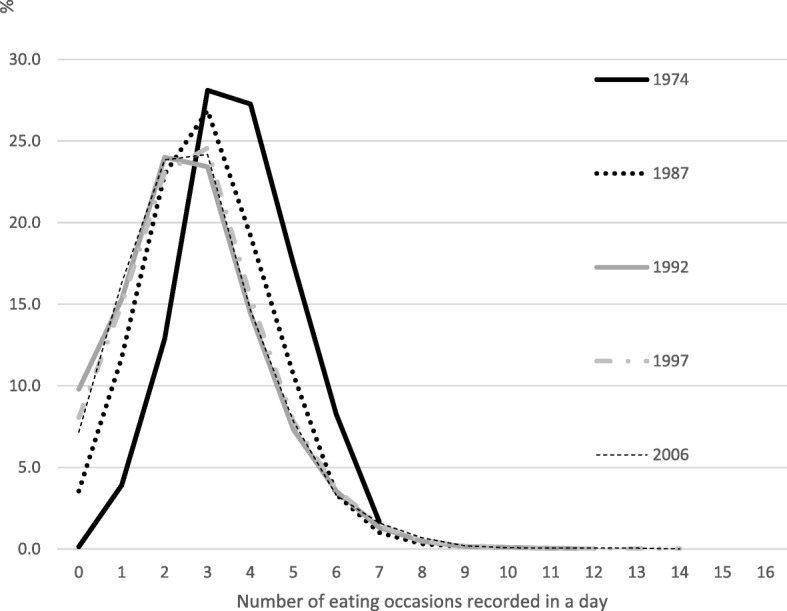
Historical change in urban Australian’s eating events as primary activity

Four decades ago, the distribution of eating events was tightly arranged around 3–4 events per day. Very few people recorded either no eating all day or ‘grazing’ most of the day. Figure 2 shows that the lines depicting eating events over the following decades have flattened and moved to the left, with a long tail to the right. In other words, eating is more dispersed, with fewer meal events and a smaller group of constant grazers. Using 1974 as the reference point, this shift was most dramatic in the late 1980s and through to 1992, but it continued at a diminishing pace into the first decade of the twenty-first century. This is why the Brazilian guidelines (led by Monteiro et al) urge people to ‘eat daily meals at similar times … avoid “snacking” [and] … eat slowly, with full attention … enjoy eating without engaging in another activity’, and eat ‘where there is no inhibition to consume unlimited amounts of food’ [21].

### Home food preparation

**Fig. 3:**
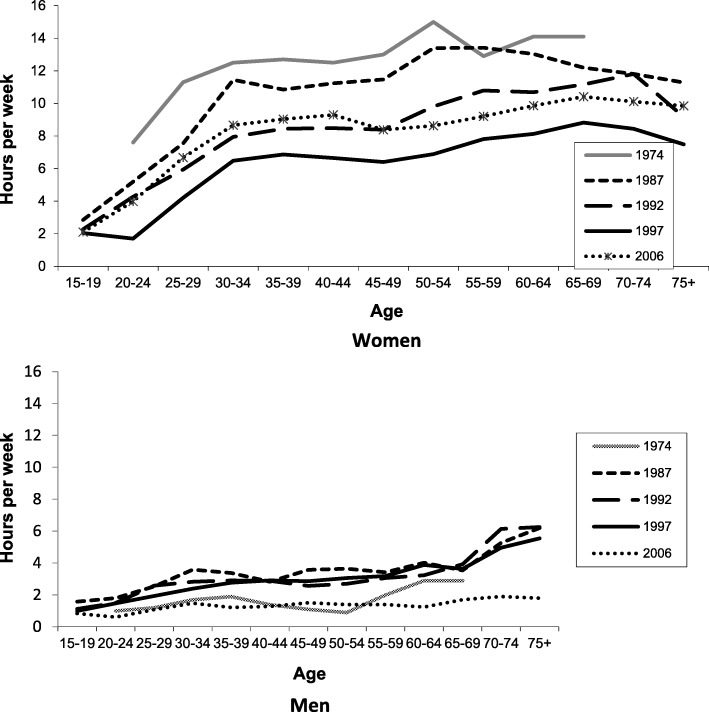
Australian adults’ time spent in home food preparation

Figure 3 shows the time spent by urban Australian men and women in home food preparation for the oldest (1974) and the most recent (2006) time-use surveys. For ease of interpretation, overall change over the period is mapped at five-year intervals. Men and women are analysed separately in each survey. They are divided into five-year age groups to control for the largest compositional change in the Australian population – ageing. There has been little change in men’s food preparation time between 1974 and 2006, but there has been a dramatic reduction in the time women spend. Australian women in their middle years in 2006 devoted five hours less per week to food preparation than in 1974, a reduction of over a third. This reduction has probably been encouraged by the mass entry of women into the workforce (especially married women), combined with the availability of market substitutes and the slight increase in husbands’ meal preparation [11]. Although rarely remarked upon, this is one of the largest social changes of the second half of the twentieth century. This change has been accompanied by greater household expenditure on market substitutes for home cooking, that is, commercially prepared, more processed foods [22].

### Eating in company

As previously mentioned, the method of collecting data in 1987 made it impossible to separate eating during paid employment from other work breaks. However, this obstacle is easily overcome by broadening the category of eating time to include all breaks at work. The effect of this adjustment is minor – the difference between the time spent eating using the strict definition of eating and the broader version is small. However, the measured historical changes in the social context of eating are not very revealing. If eating at home in the company of one’s family has declined it might be expected that eating alone would have increased [23]. However, eating alone has decreased, from 20 min per day in 1974 to 13 min in 2006. This reflects the historical pattern of decline in all time spent eating. Neither has eating alone increased as a proportion of total eating time, hovering around 20% throughout the last 32 years.

### Changes in the timing of daily eating events

**Fig. 4:**
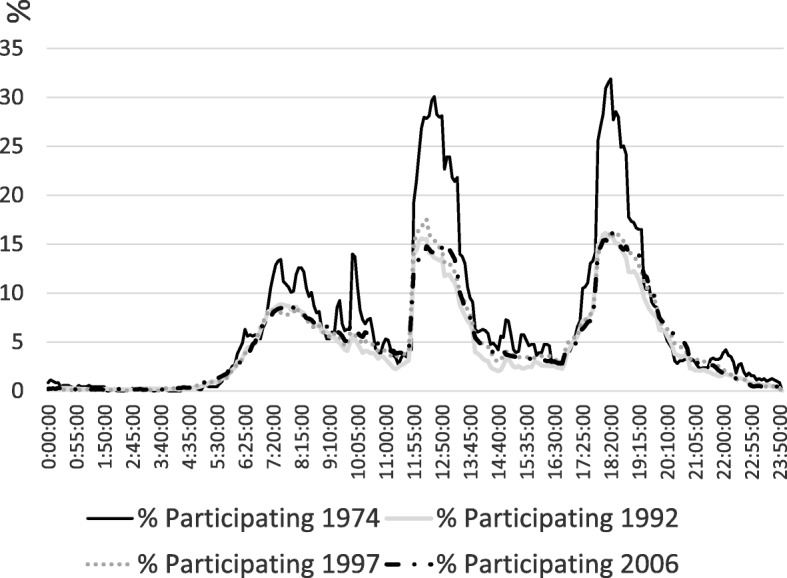
Changing timing of urban adult eating in Australia 1974 to 2006

One way of displaying the changes in the social organization of eating (and its increasing disorganization) is to plot the percentage of the population reporting eating as their main (primary) activity by time of day. Figure 4 shows that there were three daily peaks of eating in 1974 – of breakfast, lunch and dinner. (Due to incompatibilities, the data for 1987 are not shown). Breakfast in 1974 was spread over three hours 40 min (from 7:20 am to 11:00 am). Rates of participation varied between 5 and 14%, reflecting differing employment schedules and the more relaxed schedules on weekends. By 1992, although the breakfast peak exhibited the same time spread, the proportion of the population in this and subsequent surveys falls beneath the 1974 line, showing a small diminution in breakfast as a main eating activity. The decreases since 1974 in lunch and dinner are much larger. Peak participation in lunch falls from 30% in 1974 to around 15% in 1992, and stays at this level into the next century. As well, the duration of this lunch peak is shorter. The dinner peak shows a similar precipitate fall in participation, from 32% to barely 16% between 1974 and 1992. This shows both weaker social coordination of eating and a lower priority for eating at normative eating times. Over the last decades of the twentieth century, eating became less socially organized. It also became less an event shared with others, and instead an incidental and relatively unimportant activity, often undertaken as an accompaniment to other activities.

A paradox emerges from these results. At the precise historical moment when two-thirds of the adult Australian population has an overweight or obese BMI, the time spent in food consumption has diminished by a third, and there are fewer events where eating is the main activity.

There are two possible answers to this conundrum. First, if humans eat too fast they may get ahead of their body’s ability to signal satiety. Because the satiety signal arrives late, there is an overshoot in the food consumed. The alternative resolution is that, in addition to possible faster eating, eating has become so disorganized that much of it is not memorable. It slips into a realm of mindless eating and is neither recalled nor recorded in time-diaries.

## Discussion

### Estimating food intake and forgotten eating

Nutritionists have been using diary-like instruments for studying food intake for more than half a century [24, 25]. Nutritionists do use 24-h food diaries, but they often prefer records of three days or longer because what an individual eats on any particular day may be atypical. However, while three-day records are needed for the treatment of individual patients, multiple days do not increase the accuracy of population averages.

Evidence over many decades about the under-reporting of food intake demonstrates how easily subjects can deceive themselves about how often they eat. Respondents with the unhealthiest weight are the most likely to under-report eating [25, 27]. Until the appearance of doubly labelled water studies (DLW), recording food consumed and weighing it was the ‘gold standard’ for measuring the energy intake from eating but free-living subjects have found this practically impossible. Studies have confirmed the suspicion that self-reporting involves some degree of under-reporting, estimated to be in the range of 4 to 37% [25, 26, 28]. It has even cast doubt on the accuracy of weighted records as a method of estimating energy intake.

The current ‘best practice’ in epidemiological studies of energy intake is the United States Department of Agriculture’s (USDA) Automated, Multi-Pass Method (AMPM). This is a computer-administered telephone interview which collects information for the previous day, supported by a booklet for estimating portion size. It is administered in five steps: (1) a ‘quick list’ of any food and beverages consumed on the previous day; (2) prompts for forgotten food or drinks consumed; (3) recall of eating occasions; (4) a detailed description of food consumed in the last 24 h; and (5) use of the USDA-provided food booklet to estimate portion sizes [27, 29]. These five steps are followed by another reminder about forgotten foods.

USDA researchers have conducted research to validate their estimates of energy intake against DLW [30]. The findings suggest that the AMPM reduces error but does not eliminate it. This is disappointing, given that under-reporting is more serious among the overweight/obese [21]. There is a very short period between weighings (14 days). For a male who is 1.778 m (5 ft 10 in.) tall, it only takes an increase of 0.7 lb. (0.3 kg) to shift from the upper bound healthy weight BMI to overweight. Over 32 years, a weight gain of 0.5 kg a year would make a person of average height obese.

That self-reports of food intake are implausible is suggested by what Goldberg has called ‘cut-off values’ [31]. These are based on the ‘fundamental principles of energy physiology to define minimum cut-off limits for energy intake below which a person of a given sex, age and body weight could not live a normal life-style’. Using Goldberg cut-offs, a 2014 Australian Bureau of Statistics (ABS) study compared two surveys 16 years apart. It found that, although the mean body weight of the Australian population had risen over this period, there was an increase in the rate of reporting ‘implausibly’ low energy intakes [32]. In every age group (beyond the age of 10 years), the proportion of people reporting energy intake below the Goldberg cut-offs approximately doubled over that time period. There was a slightly higher proportion of females, and the proportion increased with BMI. By 2011–2012, 34% of obese males and 37% of obese females were reporting implausibly low energy intakes. This ABS study noted that even these figures probably under-estimated the actual rate of under-reporting. Citing the work of Black [33], the ABS study said that ‘Goldberg cut-off, when used in this way on a single day of intake and with overall energy expenditure from physical activity, has been estimated to find only half of all actual under-reporters’. Overall, the mean daily energy intake reported in 2011–12 was 9% lower than in 1995 [32]. This study suggests that there has been a significant growth in mindless eating over the very decades when there has been an increase in overweight/obesity.

### What kinds of eating are difficult to recall?

Another method of verifying self-reported information was used in the ‘Capture 24’ study [34]. This was designed to benchmark self-reported time diaries against ‘objective’ machine records. The machines were wearable cameras and accelerometers (the accelerometer results are not discussed here). The researchers also talked the respondents through their record of the day. Such ‘reconstruction’ interviews are often used to ensure data quality in time-use collections (for example, the pilot New Zealand Time Use survey) [35]. During the Capture 24 ‘reconstruction’ interview, the information from the camera images was used to refine the coding of the time diary. The two forms of information were compared after some adjustment for the different granularities of each data collection (10-min intervals for the diary and less than one minute between camera images). In order to deal with the small camera time intervals, diaries were coded at the two-digit levels, producing 33 activity categories derived from the Harmonized European Time-Use Survey (HETUS) activity classification.

Gershuny et al. reported that ‘the aggregate mean times in activities as recorded by the camera evidence interpreted by the experimenters, and by the participants in their self-reported diaries are in general rather similar’ [34]. Eating and television viewing were given more time than was captured by the camera, while reading and non-regular forms of work were underestimated when compared to the camera images. Gershuny at al comment that ‘if we just take the 31 two digit activities [those less than 100 minutes duration] as cases, we arrive at a correlation coefficient, between the diary and camera estimates, of 0.975 … This is a remarkably high level of association between a self-report estimate and a criterion measure’ [34].

Both the diary and the camera captured situations where more than one activity was performed at the same time. Time-diary collection asks respondents to record their ‘main’ (or ‘primary’) activity and a ‘secondary’ activity (if they ‘were doing something else at the same time’). Similarly, the ‘reconstruction interview’ required the respondent to name both activities, either as the main/primary activity or as the secondary activity (e.g. working at the computer while nibbling nuts, or viewing television while consuming snacks). Revealingly, the authors of the Capture 24 project found a substantial amount of eating as secondary activity. Almost as many minutes were spent in eating as a secondary activity as were spent in eating as a primary activity. It seems that eating is often not the focus of conscious activity and is therefore not recorded, perhaps even lost from memory. This suggests that mindless eating is a big part of contemporary food consumption, as shown in Table 2.

**Table 2:** Time-reporting hierarchy as seen in the camera record (mins/day)

Judged as	eating	tv	reading
Primary only	55	64	30
Primary + 1 secondary	108	97	42

### Further research

Preliminary results of a study by the authors indicate that changes in the patterns of eating in Australia are similar to those in other developed English-speaking countries. A preliminary inspection of French time-use data suggests that the French might be following a different path [36]. Their mean eating time increased over the same decades that saw the average eating times in English-speaking countries decreased. The French rates of overweight and obesity are similar to those in English-speaking countries before the rates of BMI began their accelerated rise. A deeper investigation of the ways the French organize their eating, using the same techniques used for Australia, could reveal some interesting public health advice about the social organization of eating.

## Conclusions

Repeated time-use survey data show that Australians have significantly altered their eating patterns since the 1970s. These changes accompany the well-known rise in the rates of adult overweight/obesity in the advanced economies, although the changes would seem to imply the opposite. The time devoted to eating as a ‘main’ activity has fallen, as has the number of eating events. There has also been a substantial decline in socially synchronized eating, especially lunch and the evening meal. However, research using objective measures of energy output (e.g. DLW), suggests that there has been an increasing trend towards under-reporting of food intake in recent decades. Overweight respondents in particular are more likely to report implausible food intakes.

The findings of the study comparing time-use diaries with the information from wearable cameras suggest that it is difficult for respondents to recall and report incidental eating, that which accompanies another ‘main’ activity such as working at a desk or watching a screen. This mindless incidental eating captured by the cameras occupies roughly the same time as memorable mindful meals. Over the last 30–40 years eating has become socially disorganized, and the disappearance of synchronized meal times may have contributed to the contemporary crisis of overweight/obesity.

A further implication is that individualized health promotion through labelling and nutritional education is not a successful strategy. It may have privatized the eating of highly processed, ‘convenience’ foods so completely that no one seems able to recall them. Ignoring the social aspects of eating may also have privatized the eating of convenience foods. The obvious next step is to perform a similar analysis of eating using the French time-use surveys for a comparable period. Strategies promoting the social synchronization of eating may be of assistance.

## Additional file

Additional file 1: **Table S1.** Australian time-use samples used. (DOCX 14 kb)

## Abbreviations

ABS: Australian Bureau of Statistics
AMPM: Automated, Multi-Pass Method
BMI: body mass index
DLW: doubly labelled water studies
HETUS: Harmonized European Time-Use Survey
MTUS: Multinational Time-Use-Study
USDA: United States Department of Agriculture

## References

[1] Murray CJ, Lopez AD (1997). Alternative projections of mortality and disability by cause 1990-2020: global burden of disease Study. Lancet.

[2] Keys A, Keys M (1960). Eat well and stay well.

[3] NCD Risk Factor Collaboration (2016). Trends in adult body-mass index in 200 countries from 1975 to 2014: a pooled analysis of 1698 population-based measurement studies with 19· 2 million participants. Lancet.

[4] Organisation for Economic Cooperation and Development [OECD] OBESITY Update, June 2014, Paris, OECD. www.oecd.org/health/Obesity-Update-2014.pdf: 2 Accessed 18/1/2018.

[5] Monteiro CA (2009). Nutrition and health. The issue is not food, nor nutrients, so much as processing. Public Health Nutr.

[6] Monteiro CA, Cannon G (2012). The impact of transnational ‘big food’ companies on the south: a view from Brazil. PLoS Med.

[7] Monteiro CA, Moubarac JC, Cannon G, Ng SW, Popkin B (2013). Ultra-processed products are becoming dominant in the global food system. Obes Rev.

[8] Monteiro CA, Levy RB, Claro RM, de Castro IRR, Cannon G (2010). Increasing consumption of ultra-processed foods and likely impact on human health: evidence from Brazil. Public Health Nutr.

[9] Schmidt MI, Duncan BB, e Silva GA, Menezes AM, Monteiro CA, Barreto SM, Chor D, Menezes PR (2011). Chronic non-communicable diseases in Brazil: burden and current challenges. Lancet.

[10] Moubarac JC, Claro RM, Baraldi LG, Levy RB, Martins APB, Cannon G, Monteiro CA (2013). International differences in cost and consumption of ready-to-consume food and drink products: United Kingdom and Brazil, 2008–2009. Global public health.

[11] Moubarac JC, Martins AP, Claro RM, Levy RB, Cannon G, Monteiro CA (2013). Consumption of ultra-processed foods and likely impact on human health. Evidence from Canada. Public Health Nutr.

[12] Moodie R, Stuckler D, Monteiro C, Sheron N, Neal B, Thamarangsi T, Lincoln P, Casswell S, Lancet NCD Action group (2013). Profits and pandemics: prevention of harmful effects of tobacco, alcohol, and ultra-processed food and drink industries. Lancet.

[13] Levy RB, Claro RM, Mondini L, Sichieri R, Monteiro CA (2012). Regional and socioeconomic distribution of household food availability in Brazil, in 2008-2009. Rev Saude Publica.

[14] Fischler C (2011). Commensality, society and culture. Soc Sci Info.

[15] Multinational Time-Use-Study: Centre for Time-Use Research, University of Oxford. https://www.timeuse.org/mtus. Accessed 18/1/2018.

[16] Australian Bureau of Statistics: New South Wales Office. Information paper: time use pilot survey. Sydney, May-June 1987. Cat*.* No*.* 4111.1.

[17] Australian Cities Commission, Time-Use Survey. 1974. https://dataverse.ada.edu.au/dataset.xhtml?persistentId=doi:10.26193/AUTMWM. Accessed 18/1/2018.

[18] Szalai A (1972). The use of time: daily activities of urban and suburban populations in twelve countries. The Hague.

[19] Robinson JP, Godbey G (1997). Time for life: the surprising ways Americans use their time. University Park.

[20] Cohen J (1992). A power primer. Psych Bull.

[21] Ministry of Health of Brazil, Dietary guidelines for the Brazilian population, 2nd edition, Brazilia, Ministry of Health, 2014, pp.92–93.

[22] Bittman M, Matheson G, Meagher G (1999). The changing boundary between home and market: Australian trends in outsourcing domestic labour. Work, Employ and Soc; 1999.

[23] Mennell, S., Murcott, A., & Van Otterloo, A. H. The sociology of food: eating, diet, and culture 1992; 40; (2). Sage Publications.

[24] Willett WC, Sampson L, Stampfer MJ, Rosner B, Bain C, Witschi J, Hennekens CH, Speizer FE (1985). Reproducibility and validity of a semiquantitative food frequency questionnaire. Am J Epidem.

[25] Thompson FE, Subar AF (2008). Dietary assessment methodology. Nutrition in the Prevention and Treatment of Disease.

[26] Kalton, G. (1985). Sample design issues in time diary studies. In F. Juster and F. Stafford (Eds.), Time, goods and well-being (pp. 93–112). Ann Arbor, MI: The University of Michigan.

[27] Young CM, Trulson MF (1960). Methodology for dietary studies in epidemiological surveys. II—strengths and weaknesses of existing methods. Am J of Pub H.

[28] Hill RJ, Davies PSW (2001). The validity of self-reported energy intake as determined using the doubly labelled water technique. Br J of Nutr.

[29] McBride J (2001). Was it a slab, a slice, or a sliver?. Agr Res.

[30] Moshfegh AJ, Rhodes DG, Baer DJ (2008). The US Department of Agriculture Automated Multiple-Pass Method reduces bias in the collection of energy intakes. Am J of Clin Nutr.

[31] Goldberg GR, Black AE, Jebb SA (1991). Critical evaluation of energy intake data using fundamental principles of energy physiology: 1 derivation of cut-off limits to identify under-reporting. Euro J of Clin Nutr.

[32] Australian Bureau of Statistics. Under-reporting in nutrition surveys. Australian Health Survey: Users’ Guide, 2011–13. 2014. Cat. No. 4363.0.55.001. http://www.abs.gov.au/ausstats/abs@.nsf/Lookup/4363.0.55.001Chapter651512011-13. Accessed 18/1/2018.

[33] Black AE (2000). The sensitivity and specificity of the Goldberg cut-off for EI:BMR for identifying diet reports of poor validity. European Journal of Clinical Nutrition.

[34] Gershuny J, Harms T, Doherty A, Thomas E, Milton K, Kelly P, Foster C. Working Paper: CAPTURE24: Testing self-report time-use diaries against objective instruments in real time: University of Oxford, Department of Sociology, Centre for Time Use Research; 2017. https://www.timeuse.org/sites/default/files/2017-10/CTUR%20WP%2010%202017_2.pdf

[35] Stats NZ. Testing Time: report of the 1990 Time Use pilot survey. Wellington; 1991.

[36] Simini B (2000). Serge Renaud: from French paradox to Cretan miracle. Lancet.

