# Supplementary material for: The social disorganization of eating: a neglected determinant of the Australian epidemic of overweight/obesity
Source: BMC Public Health. 2019 Jun 3;19(Suppl 2):454. doi: 10.1186/s12889-019-6768-3 (PMC6546620; doi:10.1186/s12889-019-6768-3)
Supplement: Supplementary file 1 — Table S1. Australian time-use samples used. (DOCX 14 kb) [file 12889_2019_6768_MOESM1_ESM.docx]

Table S1 Australian time-use samples used

| Year | Total sample (diary days, people)) | Capital city diaries used in analysis | Age range | Estimated % adult overweight/obese | % Obese |
| --- | --- | --- | --- | --- | --- |
| 1974 | 1491 diaries in total | Only 715 diaries from 715 people, Melbourne, Melbourne only used (Cities Commission) | 18-65 years | 40-43^1^ | Not available  But likely to be  < 8^6^ |
| 1987 | 3,181 diary days of information for 1,611 people | 3,181 diaries from 1,611 people, Sydney Statistical District (ABS, NSW) | 15+ years | 45^2^ | 9^7^ |
| 1992 | 13,937 diary days for 6,879 people | 8,196 diaries from 4,140 people living in major urban centres (ABS) | 15+ years | 50^3^ | 9^8^ |
| 1997 | 14,315 diary days for 8,618 people | 8,584 diaries from 4,351 people living in major urban centres (ABS) | 15+ years | 57^4^ | 17^9^ |
| 2006 | 13,732 diary days 6,961 people | 8,699 diaries from 4,395 people living in major urban centres (ABS) | 15+ years | 61^5^ | 21^10^ |

1 .Author’s own linear projection to extended OECD projection

2. OECD projection

3. OECD projection

4. Based on AIHW estimate for 1995

5. Based on AIHW estimate for 2007-8

6., 7., 8., 9., 10. Projection based on Australian National Preventive Health Agency
